# Simvastatin-induced cognitive dysfunction: two case reports

**DOI:** 10.1186/s13256-016-0877-8

**Published:** 2016-04-05

**Authors:** Chathurie Suraweera, Varuni de Silva, Raveen Hanwella

**Affiliations:** 1 University Psychological Medicine Unit, National Hospital of Sri Lanka, Colombo, Sri Lanka; 2 Department of Psychological Medicine, Faculty of Medicine, Kynsey Road, Colombo 8, Sri Lanka

**Keywords:** Simvastatin, Cognitive deficits, Memory loss

## Abstract

**Background:**

Simvastatin is commonly prescribed for hypercholesterolemia to reduce vascular risk in patients. Some of these patients have dementia with cognitive defects of several domains. Although protective effects seem to be present, there is emerging evidence that statins cause cognitive impairment.

The role of cholesterol in cognitive function is complex. This is reflected in the effects that statins show on cognition functions. The reduction in cholesterol levels seen with statins is effective in improving learning and memory in some patients. However, there is emerging evidence that statins may worsen cognitive function. Similarly, there are major concerns over whether statins alleviate or worsen cognitive problems. The correlation between cholesterol levels and cognitive function is still controversial, mainly due to a lack of robust evidence.

**Case presentation:**

We report the cases of two Asian patients who developed cognitive deficits after starting simvastatin. A 32-year-old man and a 54-year-old woman developed different but clear cognitive deficits that reversed after stopping simvastatin.

**Conclusions:**

The possibility of new-onset cognitive dysfunction and the deterioration of existing cognitive deficits should be considered when prescribing simvastatin to patients.

## Background

Simvastatin is a statin belonging to the 3-hydroxy-3-methylglutaryl coenzyme A (HMG-CoA) reductase inhibitors that lower cholesterol levels, particularly low-density lipoprotein (LDL) cholesterol and triglycerides. It increases high-density lipoprotein (HDL) cholesterol. Statins generally have minimal adverse effects, with the common side effects being myopathies, effects on liver enzymes, diarrhea, and, rarely, rhabdomyolysis [1]. Several case reports and case series have suggested a potential association between statins and cognitive impairment [2].

With the increased diagnosis of hypercholesterolemia, the prescription of statins has also increased. Because cholesterol is heavily implicated as a causative factor in the formation of amyloid plaques, a reduction in cholesterol formation is postulated to slow plaque formation [3]. Statins are also reported to possess anti-inflammatory and anti-oxidant properties. There are several studies and meta-analyses [3] confirming the benefits of statin use on cognitive impairment.

Within the population receiving statins, there is a high proportion with memory loss and associated cognitive symptoms owing to the strong correlation of vascular risk factors, like diabetes and hypercholesterolemia, with vascular and Alzheimer’s dementia. Statins are used to control vascular risk factors that may contribute to dementia, and there is emerging evidence that statins may play a protective role in improving memory problems in dementia [4, 5]. However, there is also increasing concern that statins may be a causative factor for cognitive problems, mainly memory problems [2], although this has not yet been reported as a side effect in the British National Formulary.

The postulation that statins can cause cognitive decline is based on the fact that lipophilic statins like atorvastatin and simvastatin show increased crossing of the blood–brain barrier compared to hydrophilic statins (for example, pravastatin and rosuvastatin). Two possible mechanisms are proposed: (1) the reduced availability of cholesterol caused by statins might impair the integrity of the neuronal and glial cell membrane, resulting in slowed conduction of neuronal impulses [6]; and (2) the reduced re-myelination and reduction in coenzyme Q10 levels impairs mitochondrial function and may lead to an increase in oxidative stress [7, 8]. However, in a study investigating the long-term effects of treatment with pravastatin and atorvastatin in adult rats, pravastatin tended to impair learning, implying an impact on working memory and object recognition memory that was reversible on discontinuation, whereas atorvastatin did not impair either task. This model contradicts the postulated mechanism of lipophilic statins causing more cognitive impairment because pravastatin is hydrophilic and atorvastatin is lipophilic [9]. Two randomized control trials on simvastatin [10] and pravastatin [11], which included a large number of patients, as well as a study conducted in Australia [12] did not identify a relationship between statin usage and cognitive decline. However, there are published case reports on statins causing memory loss [13].

In a study of 60 patients who had memory loss associated with statins, 36 patients received simvastatin, 23 atorvastatin, and 1 pravastatin [13]. About 50 % of patients showed cognitive adverse effects within 2 months of therapy. A problem with this study, as well as most other case reports, is the lack of formal cognitive test results in the patients who developed cognitive changes. No specific memory test results were documented in any of the 60 reports. Four reports documented tests such as computed tomography, magnetic resonance imaging (MRI), intelligence quotient (IQ), and an unidentified cognitive test. Test results were normal for three of the four patients; MRI results were unknown for one patient [13]. In a randomized trial with healthy adults, simvastatin was associated with decreased performance on some neuropsychological tests compared with the placebo [14]. A survey conducted in 171 patients on statins reported that cognitive problems associated with statins have variable onset and recovery courses, and that there is a clear relationship with the potency, resulting in a significant negative impact on quality of life [15]. In 2012, the United States Food and Drug Administration (FDA) changed labeling for statins, advising of the possibility of cognitive impairment and further adding to the concerns regarding cognitive decline [16].

We report the cases of two patients who reported memory loss and associated cognitive deficits after starting simvastatin and recovered following discontinuation. We did formal cognitive testing as well as brain imaging in both patients, which helps to rectify the lack of proper neuropsychiatric assessments in existing case studies.

## Case presentation

Our first patient was a 32-year-old Asian man with bipolar affective disorder who was prescribed 20 mg of simvastatin for hypercholesterolemia. He complained of forgetfulness resulting in significant losses to his business one month after initiation of simvastatin. He had impaired recall and memory. He did not complain of any disturbances in his memory prior to starting simvastatin, despite being known to the services for several years. The collateral history from his family confirmed that there had been significant, noticeable impairment related to short-term memory that led him to forget some major business transactions he had carried out. History from our patient and his family was obtained to rule out any possible contributory factors for his symptoms. Our patient did not have any family history of hypercholesterolemia. He had no features to suggest any vascular events in the preceding months that may have contributed to the fairly rapid development of cognitive dysfunction. Our patient had been stable in his mental state for more than 1 year at the time he developed cognitive symptoms.

Our patient did not have any abnormalities in an examination of his central nervous system and an MRI yielded normal results. Results from biochemical testing carried out to rule out possible contributory factors, including lipid profiles, were normal. His memory, as reported by our patient as well as his relatives, improved significantly after simvastatin was stopped. Although improvements were seen in his short-term recall 3 months after stopping simvastatin, the improvement was not significant on neuropsychological testing. The changes in the Montreal Cognitive Assessment (MOCA) scale, Mini Mental State Examination (MMSE), and memory component of Neuropsychiatry Unit Cognitive Assessment Tool (NUCOG) are shown in Table 1. Although improvements were observed in his cognitive profile, his cholesterol levels were uncontrolled because he refused to accept any form of treatment for hypercholesterolemia.

**Table 1:** Neuropsychological test scores during and after stopping simvastatin

Domain		With simvastatin	After stopping simvastatin
Orientation		Intact	Intact
Attention	– Forward digit span	6	7
	– Reverse digit span	4	6
Concentration (serial sevens)		Intact	Intact
Registration (5 items)		Intact	Intact
Recall (5 items)		2/5	4/5
Long-term memory		Intact	Intact
Montreal Cognitive Assessment		24/30	27/30
Mini Mental State Examination		28/30	28/30
Neuropsychiatry Unit Cognitive Assessment Tool			
Attention		19/20	20/20
Visuoconstruction		19/20	19/20
Memory		14.5/20	18/20
Executive		20/20	20/20
Language		20/20	20/20
Total		92.5/100	97/100

Our second patient was a 54-year-old Asian woman diagnosed with treatment-resistant schizophrenia who developed hypercholesterolemia while on clozapine. She developed memory impairment and difficulty executing day-to-day activities 2 months after starting simvastatin. At the time of presentation, she had had these symptoms for a year. As in the case of our first patient, there were no prior complaints of memory symptoms. Her history was obtained from her husband to rule out other possible causes for her symptoms. Our patient did not have any family history of hypercholesterolemia. Her mental state had been stable for several years prior to the development of cognitive symptoms. She did not have any neurological abnormalities on examination and her MRI was normal. No abnormalities were noted in biochemical testing, including her lipid profile. Her scores on cognitive assessment on simvastatin and 3 months after stopping simvastatin are shown in Table 2. On initial testing, there was impairment in the domains of recall, attention, visuoconstruction, memory, executive functions and language, which improved after discontinuation of simvastatin. Similar to our first patient, the observed improvement of cognitive functions as reported by our patient and her family was not reflected on formal testing. She was initiated on a non-statin lipid-regulating agent and her cholesterol levels are well controlled.

**Table 2:** Neuropsychological test scores during and after stopping simvastatin

Domain		With simvastatin	After stopping simvastatin
Orientation		Intact	Intact
Attention	– Forward digit span	5	7
	– Reverse digit span	4	6
Concentration		Intact	Intact
Registration		Intact	Intact
Recall		1/5	2/5
Long-term memory		Intact	Intact
Montreal Cognitive Assessment		18/30	21/30
Mini Mental State Examination		28/30	30/30
Neuropsychiatry Unit Cognitive Assessment			
Attention		16/20	20/20
Visuoconstruction		16/20	17/20
Memory		12/20	17/20
Executive		9/20	19/20
Language		18/20	19.5/20
Total		71/100	92.5/100

**Fig. 1:**
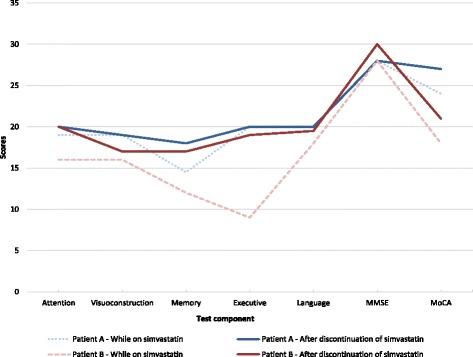
Comparison of cognitive profiles in the two patients while on simvastatin and after simvastatin was stopped

Two years after discontinuation of simvastatin, both patients remained free of any cognitive symptoms. A comparison of scores on cognitive testing of the two patients, while on simvastatin and after discontinuation, is shown in Fig. 1.

## Discussion

Although there were clear deficits in cognitive functions in our two patients, the pattern of deficits was different. Both patients had deficits in memory and recall, while one patient had deficits in attention, visuoconstruction, memory, and executive functions as well. Our case series also replicates the findings of Parker *et al*., who reported statin-related problems with memory and learning, as well as reduced performance in established cognitive tests, that resolved with discontinuation of statin therapy. The subjective and objective improvement of memory seen with their patient was similar to that in the two patients we have reported [17]. An objective improvement was seen in neuropsychological testing at least by a few points after discontinuing simvastatin. However, a common feature observed in both patients was that the observed improvement in cognitive and day-to-day functioning was significantly greater than that reflected on formal neuropsychological testing. Unfortunately, baseline neurocognitive assessments prior to the development of symptoms were unavailable in both patients because there had been no indication to carry out formal testing prior to the complaint.

Our findings are similar to several other case reports that describe development of memory and cognitive impairment. However, other cognitive deficits were not mentioned in these reports nor imaging techniques used to exclude other causes [2, 18]. A survey conducted by Evans *et al*. on 171 patients showed that cognitive problems associated with statins have a variable onset and recovery course [15]. However, both our patients developed symptoms within 2 months of starting therapy and recovered within 3 months of stopping therapy. Our findings also confirm the finding of Evans *et al*. that there is a significant negative impact on the quality of life [15] because both our patients incurred significant adverse outcomes owing to cognitive impairment, which reversed with discontinuation of the statin.

It was difficult to determine if the difference in the pattern of cognitive deficits between our two patients was due to the difference in the underlying mental disorder (bipolar affective disorder versus schizophrenia). However, it was clear that the causative factor for the cognitive deficits was not the underlying mental disorder because the deficits occurred after starting simvastatin and reversed with its discontinuation.

## Conclusions

Both our patients developed significant cognitive changes following commencement of simvastatin. These cognitive changes were different between our two patients, indicating that simvastatin may cause non-specific or global cognitive deficits, as predicted by studies done on statins. It is also important to note that the observed and perceived impairment of cognitive deterioration was greater than that reflected on formal neurological testing in both our patients. Although both patients had comorbidities, the clinical history, normal neurological and MRI findings, and the significant reversal of symptoms with discontinuation of simvastatin implicate simvastatin as the cause of our patients’ cognitive problems. Although definitive conclusions cannot be made, the possibility of new-onset as well as worsening of existing cognitive symptoms should be borne in mind when prescribing simvastatin. Further studies with detailed cognitive assessments on larger samples of patients are required to determine the exact pattern of cognitive change.

## Consent

Written informed consent was obtained from the patients for publication of this case report and any accompanying images. Copies of the written consents are available for review by the Editor-in-Chief of this journal.

## Abbreviations

FDA: US Food and Drug Administration
HDL: high-density lipoproteins
HMG-CoA reductase: 3-hydroxy-3-methylglutaryl coenzyme A reductase
IQ: intelligence quotient
LDL: low-density lipoproteins
MMSE: Mini Mental State Examination
MOCA: Montreal Cognitive Assessment Scale
MRI: magnetic resonance imaging
NUCOG: Neuropsychiatry Unit Cognitive Assessment Tool
